# How Spiritual Leadership Boosts Nurses’ Work Engagement: The Mediating Roles of Calling and Psychological Capital

**DOI:** 10.3390/ijerph17176364

**Published:** 2020-09-01

**Authors:** Wei-Li Wu, Yi-Chih Lee

**Affiliations:** Department of International Business, Chien Hsin University of Science and Technology, No.229 Jianxing Road, Taoyuan 320, Taiwan; wuweili0709@yahoo.com.tw

**Keywords:** spiritual leadership, work engagement, calling, psychological capital, nurses

## Abstract

Work engagement is an important topic in the field of nursing management. Meanwhile, spiritual leadership has been demonstrated to have a positive impact on healthcare workers. However, the relationship between spiritual leadership and work engagement is unclear. The main purpose of this study was to investigate the influence of spiritual leadership on work engagement through increased spiritual well-being and psychological capital. This study used a cross-sectional survey to collect data in Taiwan. The sample included 164 nurses, with empirical testing carried out by PROCESS Macro for SPSS. The results show that spiritual leadership has a positive influence on work engagement and that spiritual well-being (i.e., calling) and psychological capital mediate the effect of spiritual leadership on work engagement. According to the results of this study, nursing leaders must be aware of the role of spiritual leadership in promoting work engagement.

## 1. Introduction

Currently, people are being taken care of by an ever more advanced healthcare system. However, some healthcare professionals are still struggling in poor work conditions [1]. In particular, nurses, who account for a large portion of healthcare professionals, may face some unfavorable work environments, such as long work hours, high work pressure, and low wages [2]. These phenomena then cause managerial problems, for example, a high rate of intent to leave, labor shortages, and burn out in the workplace [3]. These phenomena are problematic. Nursing is an important profession, and there need to be alert, not extremely fatigued nurses in the workplace. As a result, the concept of work engagement [4] has received a lot of attention from researchers studying nursing management in the past decade [2].

Previous studies have shown that work engagement of nurses is significantly related to numerous positive outcomes [2]. For example, engaged nurses are reported to exhibit higher job satisfaction, lower burnout and job turnover intent, etc. [5,6,7]. Thus, researchers have committed to explore the antecedents of work engagement. Based on the perspective of job demands-resources (JD-R) theory, recent studies have shown that employees’ resources have a positive effect on work engagement [8]. Furthermore, according to a system review of work engagement studies on nurses, Keyko et al. (2016) [2] have developed the nursing job demands-resources (NJD-R) model for nurses’ work engagement. In the NJD-R model, the influence of leadership is an important factor related to nurses’ work engagement. Although leadership plays an important role in promoting nurses’ work engagement, most of the current studies focus only on limited leadership types, for example, authentic leadership [9,10] or transformational leadership [11]. In order to further understand the impact of leadership, other leadership styles should be taken into account in work engagement studies.

In this study, we propose that spiritual leadership will have an important impact on work engagement. Spiritual leadership is rooted in an intrinsic motivation model that comprises vision, hope/faith, and altruistic love to motivate subordinates by enhancing their spiritual well-being [12,13]. In past studies, spiritual leadership has been proven to be related to employees’ positive psychological states and reduced rates of burnout [13,14]. Increasing positive psychological states could involve expanding individuals’ personal resources, and reducing burnout rates would ameliorate the loss of human resources. Therefore, it is reasonable to assume that spiritual leadership has an impact on work engagement because the amount of resources available to an employee is the key factor determining whether or not the employee will engage with the required work [8]. More importantly, different from other leadership theories, spiritual leadership focuses more on the spiritual elements of subordinates. For example, spiritual leadership emphasizes increasing followers’ calling at work, and the nature of nursing demands highly meaningful orientation. Therefore, using spiritual leadership to study the issue of nurses’ work engagement is a suitable approach.

We develop a theoretical framework herein that draws on the NJD-R model [2] to explore how nurses’ work engagement can be explained by nurses’ perception of spiritual leadership, which helps to accumulate resources in the workplace. A core statement of the NJD-R model is that leadership can have a direct effect on work engagement; meanwhile, leadership can also influence work engagement by increasing employees’ resources. Accordingly, we propose that spiritual leadership has a direct impact on work engagement. Moreover, spiritual leadership affects work engagement by increasing personal resources (e.g., psychological capital). This study uses psychological capital to represent personal resources for the following two reasons. First, psychological capital is an important concept of personal resources [15]. Second, psychological capital has been shown to be positively related to work engagement [16].

**Figure 1:**
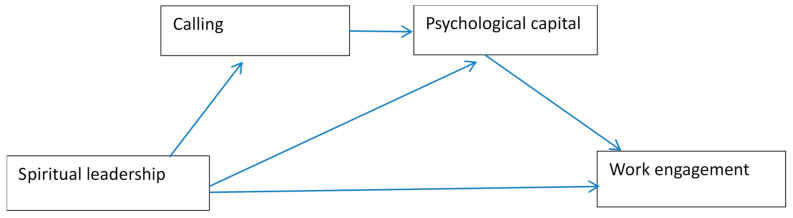
Research framework.

Moreover, this study also introduces spiritual leadership theory (SLT) [12,17], which may further explain how spiritual leadership influences psychological capital. Based on SLT, spiritual leadership affects followers by having a positive impact on their spiritual well-being. Thus, we propose that spiritual leadership will have an impact on nurses’ calling (a main dimension of spiritual well-being), thereby leading to increased psychological capital. Drawing on the NJD-R model and SLT, this study hypothesizes that spiritual leadership has positive influences on nurses’ work engagement by increasing nurses’ calling and psychological capital (Figure 1).

The results of this study are expected to make two major contributions. First, our theoretical model contributes to the literature on work engagement in the field of nursing by advancing our knowledge about the influence of spiritual leadership. Second, this study explores the mechanism operating between spiritual leadership and work engagement through an integrated view of NJD-R model and SLT. In this manner, this study enriches the content of the NJD-R model and also extends the application of SLT.

## 2. Background

### 2.1. Work Engagement and the NJD-R Model

In modern society, employees usually spend a lot of time at work where they face abundant work challenges. Work engagement is defined as “a positive, fulfilling, work-related state of mind that is characterized by vigor, dedication, and absorption” [18]. Many studies have recognized that it is important to have employees who are engaged in their work [8]. Thus, work engagement has become an important issue in the past few years. This concept originated from the field of organizational behavior. In recent years, it has also been developed significantly in the research on nursing management.

Regarding the drivers of work engagement, the JD-R model is the most commonly used theoretical perspective by which to explore the antecedents of work engagement [19]. Previous studies on nursing management have shown that some job resources [5,9] and personal resources [6,20] are significantly related to work engagement. After a rigorous literature review on work engagement in nursing studies, Keyko et al. (2016) expanded the JD-R model into the NJD-R model, a revised version; employees’ resources are still the main determinants of work engagement [2]. The main difference between these two models is that the NJD-R model is derived completely from nursing studies, and this model integrates the concept of leadership into JD-R model. Beyond the argument of the JD-R model in which employees’ resources are the main driver of work engagement, the NJD-R model emphasizes the role of leadership on developing work engagement in the field of nursing.

### 2.2. Spiritual Leadership and Work Engagement

Currently, employees typically face a lot of pressure, and many merely perform their job for the pay, without achieving enjoyment and a sense of accomplishment at work. In this context, work spirituality has gradually gained the attention of scholars [21]. In the research on work spirituality, spiritual leadership has become an important leadership issue [22]. There are three main components of spiritual leadership: vision, hope/faith, and altruistic love [12,17]. Vision is the image of a meaningful future, with some implicit or explicit commentary about why people should work hard to create their future. Hope/faith reflects the leaders’ confidence that the organization’s vision/goal/mission will be fulfilled. Altruistic love is defined “as a sense of wholeness, harmony, and well-being produced through care, concern, and appreciation for both self and others” [12]. In general, spiritual leadership could be considered as a process that intrinsically motivates followers through hope/faith in a transcendent vision of servicing others by means of altruistic love [14]. Moreover, spiritual leadership shares some common elements with religion, such as altruistic love. However, there is still a fundamental difference between spirituality and religion. Spirituality is necessary for religion, but religion is not necessary for spirituality [12]. Thus, although religion is related to spiritual leadership, it is not a necessary factor when discussing the construct of spiritual leadership.

Based on the NJD-R model, this study argues that spiritual leadership is positively related to work engagement. Spiritual leaders treat their employees with the value of altruistic love; therefore, they care for, and are concerned about, their employees in their daily work [12,17]. Moreover, spiritual leaders have strong confidence in their subordinates, encouraging them to achieve their work goals. In other words, spiritual leaders can create a supportive and friendly environment to help their employees become successful in their work. Previous studies have shown that spiritual leadership is positively related to unit productivity [14] and employees’ task performance [23]. As a result, nurses under spiritual leaders are more likely to reach work goals, while the experiences of being successful at work will lead to more engagement in their work [8]. Thus, we hypothesize the following relationship:

**Hypothsesis** **1:** *Spiritual leadership will be positively associated with nurses’ work engagement.*

### 2.3. Spiritual Leadership, Psychological Capital, and Work Engagement

Based on the NJD-R model, we contend that spiritual leadership can promote followers’ psychological capital, leading to a higher level of work engagement. Psychological capital refers to an individual’s positive psychological state of development that is characterized by self-efficacy, optimism, hope, and resiliency [24]. This study argues that employees’ psychological status can be strengthened by spiritual leadership. Spiritual leaders treat their followers with care, concern, respect, and confidence, which is likely to form a warm and caring environment [13]. This kind of supportive organizational environment is beneficial in developing employees’ psychological capital [15,25]. Previous studies have shown that the provision of workplace support is positively related to the development of psychological capital [26,27]. In addition, Chen and Li (2013) have also shown that spiritual leadership is positively related to employees’ self-concept (self-esteem and self-efficacy) [13]; self-efficacy is one of the main components of psychological capital. As a result, this study suggests that spiritual leadership is positively associated with psychological capital.

Furthermore, this study suggests that employees with psychological capital tend to engage more in their work. If employees have a higher level of psychological capital, it implies that they have more self-regard and goal orientation, causing intrinsic motivation toward their work and devoting more personal resources to deal with work challenges [8,28]. Usually, employees with high psychological capital demonstrate better job performance and experience higher levels of job satisfaction [29]. Because they experience a greater sense of achievement and feelings of job satisfaction at work, they tend to engage more in their work. Previous studies have shown that psychological capital is significantly related to work engagement [16,30]. This causal relationship could also be found in the field of nursing research [31]. To summarize the above descriptions, spiritual leadership can promote psychological capital, which is positively related to work engagement. As a result, this research proposes the following hypothesis:

**Hypothsesis** **2:** *Spiritual leadership exhibits a positive indirect relationship with work engagement via increased psychological capital.*

### 2.4. Integrating SLT in the NJD-R Model

Spiritual leadership theory was originally developed to explain how spiritual leadership affects followers’ positive performances in the workplace by increasing their spiritual well-being [12,17]. Awakening followers’ spiritual well-being is a very important influential mechanism of spiritual leadership. Based on the NJD-R models, we have only explained how spiritual leadership enhances psychological capital by establishing a friendly working environment. In this section, through the introduction of SLT, this study further explains how spiritual leadership enhances the spiritual well-being of employees and then increases psychological capital. Basically, there are two types of spiritual well-being in the SLT model: calling and membership. The motivation source of calling is directed at work, while the motivation source of membership is directed at the organization [13]. Because “work” engagement is the targeted construct that we discuss in this study, we use the construct of calling to present the concept of spiritual well-being derived from spiritual leadership, which impacts the development of individuals’ psychological capital.

When people are busy with work every day, some might consider work merely as a way to earn money or that the purpose of work is to achieve a higher position in an organization because they only think of work as a job or career [32]. However, people might also view work as a means to achieve self-fulfillment, find a purpose in life, or contribute to society. We describe these people as having experienced a sense of calling at work [12,33]. Calling describes “the experience of transcendence or how one makes a difference through service to others and in doing so, derives meaning and purpose in life” [12]. According to SLT [12,17], spiritual leaders can create a meaningful vision for their followers so that these followers can experience meaning in life, feel a sense of purpose, and make a difference. As a result, spiritual leaders can enhance their followers’ sense of calling in the workplace. Previous studies have shown that spiritual leadership is significantly related to calling [13,14,17].

Furthermore, according to SLT, calling is an important mechanism with which spiritual leaders promote employees’ positive outcomes [14,17]. In this study, we propose that individuals’ sense of calling derived from spiritual leadership has a positive impact on developing their psychological capital for the following three reasons. Firstly, research on calling indicates that the perception of calling is positively related to individuals’ well-being (e.g., life meaning or satisfaction) [34]. When people have a higher level of well-being, they tend to have a higher level of psychological capital [35]. Secondly, researchers have shown that calling is also positively related to self-efficacy [36], and self-efficacy is one of the important components of psychological capital. Finally, calling is positively related to job satisfaction [37]. When employees have a higher level of satisfaction, they tend to have a higher level of positive emotions. People with positive emotions usually tend to develop more psychological capital [38]. In sum, according to SLT, spiritual leadership promotes calling, which then increases the development of psychological capital.

**Hypothsesis** **3:** *Spiritual leadership exhibits a positive indirect relationship with psychological capital via increased calling.*

Based on the NJD-R model, this study explains how spiritual leadership affects work engagement via psychological capital (Hypothesis 2). Moreover, drawing on SLT, we propose that the perception of a calling is an important mechanism in the relationship between spiritual leadership and psychological capital (Hypothesis 3). Furthermore, by integrating SLT into the NJD-R model, we may be able to develop a clearer picture of how spiritual leadership has an impact on work engagement through calling and psychological capital. As a result, we propose that there is a serial mediation effect in the relation between spiritual leadership and work engagement.

**Hypothsesis** **4:** *Spiritual leadership exhibits a positive serial indirect relationship with work engagement via increased calling and consequently increased psychological capital.*

## 3. Methods

### 3.1. Setting

Data were collected from nurses in Taiwanese hospitals in the private sector. In Taiwan, because nurses face some serious problems at work, such as high working hours and pressure, instances of quitting or failing to engage in the job are high. It is important to understand how to use leadership to enhance nurses’ levels of work engagement. There are four kinds of medical institutions in Taiwan: clinics, district hospitals, regional hospitals, and medical centers. Of these, regional hospitals and medical centers have bigger scales, and they are also responsible for the training of doctors. Furthermore, compared to a regional hospital, a medical center has more advanced medical equipment and technology. Two medical institutions participated in this study. One was a medical center and the other was a regional hospital. In general, nurses in regional hospitals and medical centers face more challenges at work and have bigger workloads compared to those working in clinics or district hospitals. Thus, nurses in regional hospitals and medical centers are more suitable as samples for discussing the topic of work engagement.

### 3.2. Participants and Procedures

The method of collecting data was a survey with purposive sampling. Data were collected from September 2018 to February 2019. During sample collecting, each hospital had a coordinator who was in charge of questionnaire deliveries and returns. Questionnaires for nurses were developed based on previous studies and sent to the coordinators in person. The questionnaires were not assigned any identification to ensure that participants could complete them honestly. The coordinators recruited nurses to complete the questionnaires on a voluntary basis. After the nurses filled out the questionnaires, they gave them directly to the coordinator of the hospital. A total of 200 nurses from these two hospitals were invited to complete the questionnaires. Ultimately, we received 175 questionnaires. After excluding incomplete questionnaires, 164 usable ones remained (82% response rate).

Of the 164 nurses, 99.4% were female, and 20.7% were married. The average age was 27.23 years (SD = 7.32), the average tenure was 5.97 years (SD = 5.62), and 98.1% of the nurses had an associate’s degree or above.

### 3.3. Measures

A seven-point scale was used for all of the measurements. The response options were from 1 = “strongly disagree” to 7 = “strongly agree”. All the scale items were translated into Chinese and then translated back to ensure that the meanings of items in the Chinese version were the same as the original items. The Institutional Review Board of the hospital reviewed and approved this study.

#### 3.3.1. Spiritual Leadership

Spiritual leadership was assessed by the nurses using 17 items adopted from Fry et al. (2005) [17]. This construct included three dimensions: vision, hope/faith, and altruistic love. Sample items included the following: “I understand and am committed to my organization’s vision”, “I always do my best in my work because I have faith in my organization and its leaders”, and “My organization really cares about its people”. The Cronbach’s α for this scale was 0.98.

#### 3.3.2. Calling

Calling was rated by the nurses using four items adopted from Fry et al. (2005) [17], such as the following: “The work I do is very important to me” and “My job activities are personally meaningful to me”. The Cronbach’s α for this scale was 0.93.

#### 3.3.3. Psychological Capital

Because psychological capital is a latent construct and a reflective measurement model, it means that the indicators of this construct are highly intercorrelated and inter-exchangeable [39]. In order to reduce the participants’ burden of rating the whole questionnaire, we chose one item from each of the four dimensions (self-efficacy, optimism, hope, and resiliency) of psychological capital developed by Luthans et al. (2007) to measure this construct [24]. There were four items for the measurement of psychological capital in this study. This method of dealing with the reflective measurement model can also be found in previous studies [40,41]. Sample items included the following: “I would think of many methods to complete current work objectives” and “I feel confident in representing my work area in meetings with management”. The Cronbach’s α for this scale was 0.91.

#### 3.3.4. Work Engagement

Work engagement was rated by the nurses using nine items adopted from Schaufeli, Bakker, and Salanova (2006) [42], such as the following: “At my work, I feel bursting with energy” and “At my job, I feel strong and vigorous”. The Cronbach’s α for this scale was 0.95.

#### 3.3.5. Control Variables

This study used nurses’ demographic variables, such as age, education, and working tenure, as the control variables. We measured nurses’ age, education, and number of working years.

### 3.4. Analysis Strategies 

This study used the SPSS package to test all the hypotheses. We employed a liner regression to test Hypothesis 1. PROCESS Macro for SPSS developed by Hayes (2013) [43] was then used to verify the mediation effects, including Hypotheses 2–4. We employed PROCESS Model 4 to analyze the simple mediation effect described by Hypotheses 2 and 3 and PROCESS Model 6 to test the serial mediation effect described by Hypothesis 4. During all the testing of the PROCESS models, we used 10,000 bootstrap samples with 95% confidence intervals for the bootstrap analyses. In addition, age, education, and working tenure were controlled throughout these analyses.

## 4. Results

This study employed a four-factor confirmatory factor analysis (CFA) model for the key measurements (spiritual leadership, calling, psychological capital, and work engagement). Item parceling was used in the model [44] in order to maintain a reasonable number of degrees of freedom. The CFA model achieved an acceptable fit: CFI = 0.96, PGFI = 0.54, NFI = 0.94, IFI = 0.96, and AGFI = 0.82. All of the measurements had composite reliability above 0.90 and average variance extracted (AVE) above 0.70. The square roots of all AVE scores were higher than any correlations of possible focal pair measures. Thus, both convergent validity and discriminant validity were supported. In addition, because the four key variables were self-reported measurements from the same nurses, this study asked participants to complete the measurement of work engagement first and then fill out the measurements of mediator and independent variables later to reduce the potential effects of common method bias (CMV) in this study. Moreover, this study conducted Harman’s one-factor test [45] to analyze the potential issue of CMV bias. The result showed no serious problem regarding CMV in this study.

Table 1 provides the means, standard deviations, reliabilities, and correlations of the variables used in this study.

Hypothesis 1 predicts that spiritual leadership has a positive effect on work engagement. Model 1 of Table 2 shows that spiritual leadership was positively and significantly related to work engagement (b = 0.73, *p* < 0.001); thus, Hypothesis 1 was supported. Hypothesis 2 predicts that spiritual leadership has a positive indirect effect on work engagement through psychological capital. Model 1 of Table 3 shows that spiritual leadership was significantly and positively related to psychological capital (b = 0.70, *p* < 0.001). Model 2 of Table 3 reveals that psychological capital was significantly and positively related to work engagement (b = 0.62, *p* < 0.001). The bootstrapping analyses showed that the indirect effect of spiritual leadership on work engagement via psychological capital was 0.44, and the 95% confidence interval did not contain zero (CI = {0.3013, 0.5910}). Thus, Hypothesis 2 was supported.

Hypothesis 3 proposes that spiritual leadership has a positive indirect effect on psychological capital through calling. Model 1 of Table 4 reveals that spiritual leadership was significantly and positively related to calling (b = 0.71, *p* < 0.001). Model 2 of Table 4 shows that calling was significantly and positively related to psychological capital (b = 0.43, *p* < 0.001). The bootstrapping analyses demonstrated that the indirect effect of spiritual leadership on psychological capital via calling was 0.31, and the 95% confidence interval did not contain zero (CI = [0.2067, 0.4197]). Therefore, Hypothesis 3 was supported.

**Figure 2:**
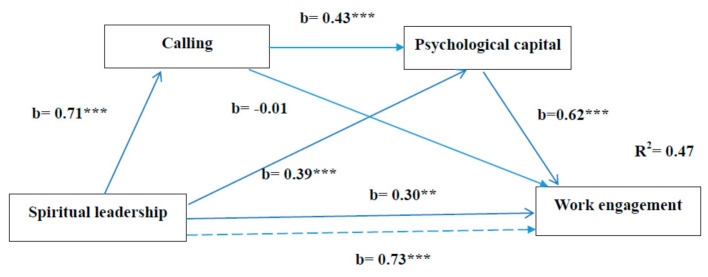
Serial mediation of calling and psychological capital. The results are based on PROCESS Model 6. Beta is not standardized coefficients. * *p* < 0.05, ** *p* < 0.01, *** *p* < 0.001. Solid lines represent direct effects. The dotted line represents the total effect.

Hypothesis 4 predicts that spiritual leadership has a positive influence on work engagement via calling and psychological capital. For this hypothesis, spiritual leadership was assumed to influence calling and psychological capital in a serial way and then have an effect on work engagement. The results of PROCESS Model 6 are presented in Figure 2. Regarding the control variables, because only working tenure had an impact on work engagement (b = 0.04, *p* < 0.05), the influences of control variables were omitted in Figure 2 in order to keep the figure simplified. As shown in Figure 2, the total effect of spiritual leadership on work engagement was at a significant level (b = 0.73, *p* < 0.001). In addition, spiritual leadership was significantly and positively related to calling (b = 0.71, *p* < 0.001); calling was significantly and positively related to psychological capital (b = 0.43, *p* < 0.001); psychological capital was significantly and positively related to work engagement (b = 0.62, *p* < 0.001). The bootstrapping analyses show that the indirect effect of spiritual leadership on work engagement via calling and psychological capital was 0.19, and the 95% confidence interval did not contain zero (CI = [0.1126, 0.3028]). As a result, Hypothesis 4 was supported.

## 5. Discussion

By investigating the association between spiritual leadership and work engagement through calling and psychological capital, this research has made several theoretical contributions. First, this study contributes to the literature on work engagement in nursing by introducing the concept of spiritual leadership. Although prior research has shown that some positive types of leadership (e.g., authentic leadership and transformational leadership) could have a positive influence on work engagement [9,11], there have been few studies examining connections between these leadership styles and work engagement through nurses’ spiritual elements. The results of this study indicate that spiritual leadership could have a significantly positive influence on work engagement. It shows that work engagement could be increased by meeting the spiritual elements of nurses. Future studies could explore other drivers of spiritual elements besides spiritual leadership, such as workplace spirituality [46], which already has a rich literature foundation.

Second, this study also supports the usefulness of the NJD-R model [2]. One of the key arguments based on the NJD-R model is that leadership is an important factor having a direct or indirect impact on work engagement, and the indirect effect mainly operates by promoting nurses’ resources. This study demonstrates that spiritual leadership not only has a direct effect on work engagement, but also has an indirect influence on work engagement via increased psychological capital, which is an important personal resource to promote work engagement according to past research [16]. Thus, the results of this study have responded to and supported the argument of the NJD-R model. Moreover, according to the perspective of the NJD-R model, there are three kinds of resources for nurses: job, professionalism, and personal resources. All of these resources have a positive influence on work engagement. Future studies could investigate how spiritual leadership affects work engagement by increasing nurses’ different kinds of resources.

Third, by integrating SLT into the NJD-R model, this study examines spiritual leadership on work engagement via calling and psychological capital. This serial mediation model helps us to have a clearer understanding of the mechanism between spiritual leadership and work engagement. More importantly, this study shows that once nurses’ spiritual well-being (i.e., calling) is satisfied by spiritual leaders, they could develop more psychological capital, which helps to enhance work engagement. Identifying the mediator of calling is vital because it suggests the importance of spiritual well-being in the workplace. This result echoes past research that emphasizes the importance of fulfilling employees’ spiritual well-being in the workplace, and it could benefit the employees and organizations [13,14,47].

Finally, although the main contribution of this study is to the literature on work engagement in nursing, we also theorize that spiritual leadership could gradually lead to the development of personal resources (i.e., psychological capital) and work engagement. Therefore, this study also contributes to the growing literature on spiritual leadership by illustrating psychological capital and work engagement as two outcomes of spiritual leadership. In the prior research on SLT, the end outcomes of spiritual leadership were usually performance, behavioral outcomes (e.g., organizational citizenship behavior), or organizational commitment [13,17,47]. This study adds a new kind of outcome, namely positive psychological resources, into the model of SLT. Both psychological capital and work engagement are important positive psychological resources in the literature on positive organizational behavior [15,48]. As a result, this study expands the breadth of the theoretical model of SLT.

The results of this study have several important implications for managers in nursing. First, the findings suggest that spiritual leadership has a direct and indirect effect on nurses’ work engagement. In other words, if leaders could adopt spiritual leadership with respect to their subordinates, it would increase their subordinates’ work engagement. Hence, managers should pay more attention to human resource practices designed for developing spiritual leadership, such as selection and training programs. Second, the relationship between nurses’ psychological capital and work engagement is confirmed. Managers can also use some training programs or interventions to increase nurses’ psychological capital. Prior research has proven that employees’ psychological capital could be promoted by workplace intervention [49,50]. Finally, our research shows that nurses’ sense of calling is an important mediator that translates spiritual leadership into psychological capital. Calling has a positive influence on psychological capital. In order to increase nurses’ sense of calling, managers should create a culture or climate that values nurses’ work. In this way, nurses will realize that they are doing a very meaningful job, which would help develop their sense of calling. In addition, managers should use reward systems carefully. If managers emphasize monetary rewards too much, nurses’ perception of the purpose of nursing work might become that of pursuing money, while overlooking the significance of nursing work itself. In this situation, the sense of calling might gradually decrease, which is detrimental for the development of psychological capital, and thus lead to a lower level of work engagement.

There are several limitations in the current study that should be addressed. First, because all of the key constructs in this study belong to an individual’s personal perceptions or psychological characteristics, we needed to ask participants to answer all of the measurement items by themselves. This self-report method might have caused the problem of CMV, although this study employed some methods of reducing this potential problem, and the statistic test for CMV was also acceptable. However, we encourage future studies to develop ways to avoid this potential problem. For example, future studies might use experiments to test the hypotheses instead of using the survey method. Second, all the hypotheses in this study imply causality. However, we collected all of the measurement data at the same point in time. Future studies could try to collect the data with a longitudinal design. Third, we used nurses from a regional hospital and a medical center as the sample for this study. Although nurses in these two types of medical institution are suitable for testing our theoretical framework, it might be better for our sample to include nurses in clinics and district hospitals as well. Furthermore, because we could not acquire information about the population of nurses in our survey hospitals, the sample used in this study may not be representative. Although this study has provided initial evidence for the relationship between spiritual leadership and work engagement, the representativeness of the sample could be improved. In order to achieve better generalization of the research results, future studies should collect a sample of nurses from all different types of hospitals and collect the sample with better sampling methods. Finally, according to the NJD-R model, the influence of nurses’ resources on work engagement might differ depending on the nurses’ job demands. However, in this study, we mainly focused on exploring the relationship between spiritual leadership and work engagement and did not discuss the potential moderating effects caused by job demands. When future studies investigate the relationships among leadership, nurses’ resources, and work engagement, they could use some constructs from job demands (such as work pressure or emotional demands) as moderators to develop a more sophisticated and vivid theoretical model of work engagement.

## 6. Conclusions

Drawing on the NJD-R model and SLT, this study makes valuable contributions to the literature on leadership and work engagement in nursing. In particular, this study discusses the relationship between leadership and work engagement from a spiritual aspect, which is a new and important perspective in nursing. Our research highlights mediators, namely calling and psychological capital, in the relationship between spiritual leadership and work engagement. This current study paints a clearer picture of the complex mechanism between spiritual leadership and work engagement. Moreover, the results of this study show that the NJD-R model offers a useful theoretical perspective on nurses’ work engagement for future studies.

## Figures and Tables

**Table 1:** Means, standard deviations, reliabilities, and correlations.

	Mean	SD	1	2	3	4	5	6	7
1. **Age**	27.25	7.18							
2. **Education ^a^**	4.51	0.55	0.21 **						
3. **Working tenure**	5.97	5.62	0.78 ***	−0.11					
4. **Spiritual leadership**	4.57	1.11	−0.37 ***	−0.20 *	−	(0.98)			
5. **Calling**	5.05	1.07	−0.28 ***	−0.12	−0.13	0.74 ***	(0.93)		
6. **Psychological capital**	4.80	1.01	−0.23 **	−0.16 *	−0.06	0.76 ***	0.78 ***	(0.91)	
7. **Work engagement**	4.01	1.27	−0.21 **	−0.23 **	0.01	0.70 ***	0.59 ***	0.72 ***	(0.95)

Reliabilities are on the diagonal parentheses. * *p* < 0.05, ** *p* < 0.01, *** *p* < 0.001, ^a^ 1 = primary school, 2 = junior high school, 3 = senior high school, 4 = associate’s degree, 5 = bachelor’s degree, 6 = master’s degree and above.

**Table 2:** Result of regression analysis.

	Work Engagement
	Model 1
**Variables**	
**Age**	−0.03
**Education**	−0.07
**Working tenure**	0.06 *
**Spiritual leadership**	0.73 ***
**R^2^**	0.47
**F**	34.98 ***

Beta is not standardized coefficients. * *p* < 0.01, ** *p* < 0.01, *** *p* < 0.001.

**Table 3:** Simple mediation analysis of the indirect effect between spiritual leadership and work engagement via psychological capital.

	Psychological Capital		Work Engagement	
	Model 1		Model 2	
	Coefficient	95% CI	Coefficient	95% CI
**Variables**				
**Age**	−0.01	−0.0354, 0.0182	−0.03	−0.0610, 0.0087
**Education**	0.04	−0.1732, 0.2465	−0.09	−0.3601, 0.1853
**Working tenure**	0.03	−0.0061, 0.0588	0.04 *	0.0022, 0.0873
**Spiritual leadership**	0.70 ***	0.6016, 0.7995	0.30 **	0.1037, 0.4876
**Psychological capital**			0.62 ***	0.4176, 0.8246
**R^2^**	0.59		0.57	
**F**	56.67 ***		41.47 ***	

The results are based on PROCESS Model 4. CI = confidence interval. Beta is not standardized coefficients. * *p* < 0.05, ** *p* < 0.01, *** *p* < 0.001.

**Table 4:** Simple mediation analysis of the indirect effect between spiritual leadership and psychological capital via calling.

	Calling		Psychological Capital	
	Model 1		Model 2	
	Coefficient	95% CI	Coefficient	95% CI
**Variables**				
**Age**	−0.01	−0.0438, 0.0163	0.00	−0.0262, 0.0209
**Education**	0.10	−0.1344, 0.3361	−0.01	−0.1915, 0.1777
**Working tenure**	0.02	−0.0191, 0.0537	0.02	−0.0097, 0.0475
**Spiritual leadership**	0.71 ***	0.6001, 0.8220	0.39 ***	0.2705, 0.5167
**Calling**			0.43 ***	0.3091, 0.5544
**R^2^**	0.55		0.69	
**F**	48.74 ***		69.55 ***	

The results are based on PROCESS Model 4. CI = confidence interval. Beta is not standardized coefficients. * *p* < 0.05, ** *p* < 0.01, *** *p* < 0.001.
